# Host–Pathogen Interaction Involved in *Trypanosoma cruzi* Infection

**DOI:** 10.3390/pathogens11050540

**Published:** 2022-05-04

**Authors:** Christian Castillo, Ulrike Kemmerling

**Affiliations:** 1 Instituto de Ciencias Biomédicas, Facultad de Medicina, Universidad de Chile, Santiago 8380453, Chile; ccastillor@uchile.cl; 2 Facultad de Medicina Veterinaria y Agronomía, Universidad de Las Américas, Santiago 7500975, Chile

Chagas disease, or American trypanosomiasis, caused by the protozoan parasite *Trypanosoma cruzi* (*T. cruzi*), is a silent, underdiagnosed, and life-threatening disease. Moreover, it constitutes a significant public health issue in Latin America and worldwide.

The probability of acquiring the disease depends on multiple factors: the parasite, the vectors, the mammalian hosts (including human beings), and socioeconomic aspects. In addition, the host–pathogen interaction is a key factor determining the probability of infection and the establishment of the disease. Thus, studying these different aspects is essential to understanding the pathogenesis of the disease and improving the diagnostic and therapeutic tools for *T. cruzi* infection. Therefore, several articles address these issues in the Special Issue “Host-Pathogen Interaction Involved in *T. cruzi* Infection” in *Pathogens*.

A state-of-the-art review of molecular and clinical aspects of chronic manifestation in Chagas disease is part of the Special Issue [1]. This review addresses the parasite’s life cycle, genetic diversity, virulence factors, infective mechanisms, and the epidemiology, clinical presentation, diagnosis, and treatment options of the principal chronic complications of Chagas disease. 

In addition, a review of the liver and its immune responses against *T. cruzi* infection is also part of the Special Issue [2]. Here, the authors present the first paper published approaching the liver’s participation in parasite infection and subsequent articles published in the last century, including recently published results. It is proposed that, after infection, activated peripheral T lymphocytes reach the liver and induce a shift to a pro-inflammatory ambient environment. Thus, there is an immunological integration and cooperation between peripheral and hepatic immunity, contributing to disease control.

This Special Issue includes several original articles. A study about the role of the muscarinic acetylcholine receptor on mice behavior and brain lesions induced by *T. cruzi* is included [3]. Thus, the muscarinic cholinergic pathway seems to be involved in immune-mediated cell invasion events, while its blockade favors infection, brain lesions, and behavioral alterations. Furthermore, since acute chagasic encephalitis is a clinically severe central nervous system manifestation, this paper contributes to the knowledge of the nervous form of Chagas disease. 

Another original paper is about congenital Chagas disease, responsible for 22.5% of new cases each year. Interestingly, placental transmission occurs in only 5% of infected mothers, and it has been proposed that the epithelial turnover of the trophoblast can be considered a local placental defense against the parasite and imply a critical control of gene expression that is regulated, among other mechanisms, by small non-coding RNAs such as microRNAs. A paper regarding *T. cruzi-*induced trophoblast epithelial turnover shows that placenta-specific miR-512-3p and miR-515-5p at least partially mediate trophoblast differentiation [4]. In addition, both miRNAs determine placental susceptibility to *ex vivo* infection of human placental explants. Knowledge about the role of parasite-modulated microRNAs in the placenta might enable their use as biomarkers as prognostic and therapeutic tools for congenital Chagas disease.

Moreover, studies about insect vectors are part of the Special Issue. Transmission patterns of vector-borne diseases are known to respond to habitat changes adaptively; as such, a study evaluated how the physical characteristics of *Triatoma dimidiata* would vary in relation to land use in El Salvador [5]. The authors suggest that mitigation studies of Chagas disease should exploit the relationships between anthropogenic land use and *T. dimidiata* morphology to evaluate how the transmission pattern of *T. cruzi* and Chagas disease symptomatology are impacted.

Another study focuses on understanding the blood meal patterns of insects that are vectors for *T. cruzi*. Knowledge about transmission dynamics is crucial for developing strategies to impede or decrease human–vector contact [6]. Thus, the authors determined the diversity of hosts that serve as blood sources for triatomines in domestic, peridomestic, and sylvatic transmission cycles in two endemic areas of Ecuador. The results showed that humans are the primary food source for triatomines, indicating that human–vector contact is more frequent than previously thought. On the other hand, the diversity of blood sources might indicate a preference driven by triatomine species. Moreover, more than one source of blood in triatomines collected in the same place indicates that dispersal of vectors occurs regardless of food availability. Therefore, the dispersal capacity of triatomines needs to be evaluated to propose an effective strategy that limits human–vector contact and, in consequence, decreases the risk of *T. cruzi* transmission.
